# MEK inhibitor, TAK-733 reduces proliferation, affects cell cycle and apoptosis, and synergizes with other targeted therapies in multiple myeloma

**DOI:** 10.1038/bcj.2016.7

**Published:** 2016-02-26

**Authors:** P de la Puente, B Muz, A Jin, F Azab, M Luderer, N N Salama, A K Azab

**Affiliations:** 1 Department of Radiation Oncology, Cancer Biology Division, Washington University in Saint Louis School of Medicine, St Louis, MO, USA; 2 Department of Pharmaceutical and Administrative Sciences, St Louis College of Pharmacy, St Louis, MO, USA; 3 Department of Pharmaceutics and Industrial Pharmacy, Cairo University Faculty of Pharmacy, Cairo, MO, Egypt

The RAS/RAF/MEK (mitogen-activated protein kinase)/ERK (extracellular-signal-regulated kinase) pathway is one of the main signaling systems that manage proliferation, differentiation and cell survival, as well as cell cycle and apoptosis.^1^ In this pathway, a cascade of phosphorylation events affects three key kinases: RAF, MEK (MAPK kinase) and ERK (MAP kinase). While MEK is not frequently mutated in human cancer, aberrant expression of MEK is observed in many different cancers.^1, 2^

Multiple myeloma (MM) is the second most common hematological malignancy with a 45% five-year survival rate of patients based on the 2004–2010 myeloma cancer statistics from SEER (Surveillance, Epidemiology, and End Results Program).^3^ The RAS/RAF/MEK/ERK pathway is often deregulated in MM cells and the prevalence of RAS mutations in myeloma is reported to be about 20–40%.^4, 5^ MEK is a serine/threonine kinase responsible for the phosphorylation of ERK1/2. ERK1/2 are unique targets of MEK, making MEK an interesting target for anticancer therapeutics.^6^ An advantage of targeting MEK is that RAF/MEK/ERK pathway is a conjunction point where several upstream signaling pathways can be blocked with the inhibition of a single kinase, MEK. MEK1/2 inhibition against MM cells has been probed to affect progression, cell cycle and apoptosis, as well as synergizes with other anti-MM agents.^7, 8^

TAK-733 is a potent and selective MEK allosteric site inhibitor for MEK, which inhibits growth *in vitro* of a broad range of cell lines. In mouse xenograft studies, TAK-733 has demonstrated suppression of tumor growth in a wide range of tumor types.^9^ Recently, more clinically relevant *in vivo* murine models of melanoma have demonstrated robust tumor growth inhibition by TAK-733.^10^ However, to date TAK-733 has not been tested in multiple myeloma. In the present study, we are investigating the role of TAK-733-mediated MEK inhibition on the progression of MM, and will test its effect on survival, cell cycle, apoptosis, sensitivity to other targeted drugs in MM cells, as well as interaction with the bone marrow (BM) microenvironment.

To investigate the effect of TAK-733 on survival, cell cycle and apoptosis, MM cell lines were cultured with the MEK inhibitor TAK-733 for 48 h. The MEK inhibitor TAK-733 reduced the proliferation of MM cell lines with IC50 values in the μM range (2–5 μM) after 48 h treatment as a single agent (Figure 1ai) and did not affect the proliferation of peripheral blood mononuclear cells from three healthy donors (Figure 1aii) measured by 3-(4,5-dimethylthiazol-2-Yl)-2,5-diphenyltetrazolium bromide (MTT) assay. Then, we confirmed the effect of TAK-733 in H929 cell proliferation and associated cell signaling by immunoblotting. We found that TAK-733 showed high potency in suppressing pERK1/2, a downstream target of MEK. While pAKT was not affected, we detected a decrease in expression of other downstream proteins involved in survival and proliferation, including pS6R and pGSK3 (Figure 1aiii). TAK-733 inhibited cell proliferation of H929 and U266 by increasing the percentage of cells in G0/G1 (enhanced G0/G1 peak) and decreased the percentage of cells in G2/M and S phase (Figure 1bi), which was confirmed by specific signaling with reduced transition cell cycle proteins (pRb and Cyclin E) (Figure 1bii). We also found that MEK inhibition increased significantly early (Annexin+/PI−), late apoptosis (Annexin+/PI+), and cell death (Annexin−/PI+) observed in two cell lines (Figure 1ci). Apoptosis was further confirmed by immunoblotting showing induction of Caspase-3 and poly(ADP-ribose) polymerase cleavage (Figure 1cii).

Although the introduction of bortezomib, a novel first-in-class proteasome inhibitor, had been a major advancement in the treatment of MM, several studies showed that 60% of patients will develop bortezomib resistance.^11^ In addition, simultaneous blockade of MEK and PI3K exhibited a synergistic effect by decreasing proliferation in solid tumors.^12^ We have recently showed that a novel specific PI3K-alpha inhibitor (BYL719) reduced proliferation, inhibited the cell cycle and induced apoptosis in MM cells.^13^ We therefore examined the combinatory effect of TAK-733 with bortezomib and BYL719 after 48 h treatment. It was found that the combination of TAK-733 with bortezomib (Figure 2ai) or BYL719 (Figure 2aii) decreased the surviving fraction of H929 cells more than either the drug alone measured by MTT. While TAK-733/bortezomib combinations showed synergistic effect only at the higher percentages of inhibition (>0.70) (Figure 2ai-inset), TAK-733/BYL719 combinations showed synergistic effect at all the combinations (Figure 2aii-inset) as shown in the CI graphs from CompuSyn software (Paramus, NJ, USA). These results provide confirmation of TAK-733 as anti-myeloma agent alone or in combination with other targeted drugs.

Some of the previous findings have been reported for other MEK inhibitors. However, to date MEK inhibitors have not been tested on a more physiologically relevant culture model, such as 3D tissue engineering bone marrow (3DTEBM) model.^14^ H929 cells were cultured either in 2D or 3DTEBM cultures alone or in co-cultured with BM stromal cells (BMSCs) derived from MM patients, cells were treated for 48 h, and then cell survival was measured by flow cytometry. TAK-733 showed that 2D co-culture of MM cells with stroma decreased the sensitivity of MM cells to TAK-733, and while monocultures on MM cells in 3D cultures decreased the sensitivity of MM cells to TAK-733, co-culture in the 3DTEBM induced the most profound resistance to TAK-733 by killing only 10 and 15% of the MM for 2.5 and 5 μM, respectively (Figure 2b). These results emphasize the role of the BM microenvironment in the sensitivity of MM cells to MEK inhibitors, which needs further evaluation, especially in the 3D context.

To overcome resistance to therapy induced by BMSCs, we combined TAK-733 with AMD3100, a CXCR4 inhibitor, which showed disruption of the interaction of MM cells, allowing improvement in therapeutic efficacy.^15^ TAK-733 was analyzed in combination with AMD3100 in mono-culture or co-culture with BMSCs for 48 h and the proliferation was analyzed by MTT assay. In Figure 2ci, AMD3100 does not affect MM cell survival and does not induce an additional effect in combination with TAK-733. However, in presence of BMSCs, combination of TAK-733 and AMD3100 overcomes the drug resistance derived from co-culture conditions (Figure 2cii). These results provide confirmation of TAK-733 as anti-myeloma agent in combination with AMD3100.

In addition, several studies have indicated that bone marrow of MM patients is hypoxic and contributes to cancer progression and hypoxia-mediated drug resistance.^16, 17, 18^ We investigated the effect of hypoxia on MM cell proliferation in the presence of TAK-733. MM cells were treated and cultured for 48 h in hypoxic and normoxic conditions and the proliferation was analyzed by MTT assay. In normoxia, TAK-733 showed 25, 60 and 90% killing for 1, 2 and 4 μM, respectively. However, hypoxia induced resistance at the higher concentrations with 45 and 70% killing for 2 and 4 μM, respectively (Figure 2d). These results on hypoxia-induced drug resistance correlate with previously reported drug resistance to other drugs.^19^

In summary, MEK plays a critical role in MM proliferation, cell survival, as well as cell cycle and apoptosis. MEK inhibition reduces proliferation and induces cell cycle arrest and apoptosis in MM cells. Furthermore, we showed TAK-733's synergistic effect with proteasome and PI3K inhibitors. Drug resistance was detected in case of co-cultures with BMSCs, hypoxia and more physiological relevant 3DTEBM cultures. However, combination with a CXCR4 inhibitor and AMD3100, overcame that drug resistance. These results provide confirmation of TAK-733 as anti-myeloma agent alone or in combination with other targeted drugs.

## Figures and Tables

**Figure 1:**
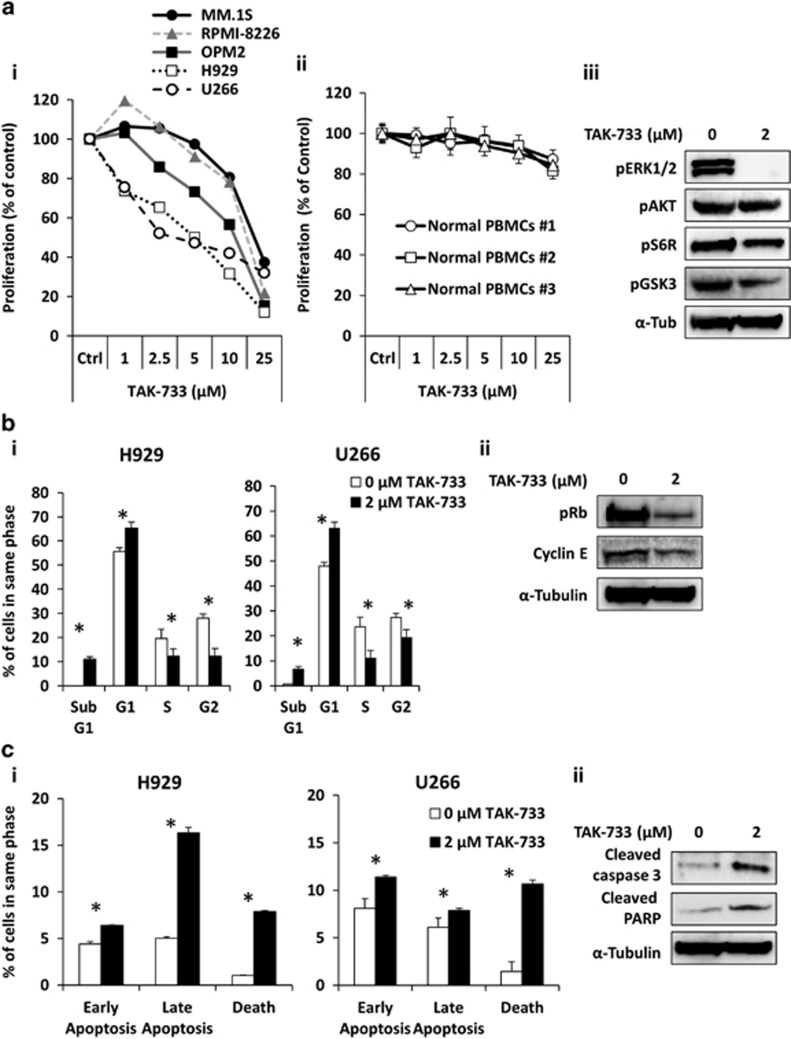
TAK-733 reduces MM proliferation, affects cell cycle and apoptosis. (**a**) The effect of TAK-733, MEK inhibitor (0-25 μM) on proliferation of MM.1S, U266, H929, RPMI-8826 and OPM2 cells (i), or normal Peripheral blood mononuclear cells (ii) for 48 h analyzed by MTT, and effect of TAK-733 (0, 2 μM) for 48 h on proliferation signaling of pERK, pAKT, pS6R and pGSK3 in H929 cells by Western blotting. (**b**) Cell cycle analysis of TAK-733 (0, 2 μM) in H929 and U266 cells for 48 h representing percent of population in each cell phase (i), and the effect of treatment for 48 h on cell cycle signaling pRb and Cyclin E in H929 cells by Western blotting (ii), (*) *P*<0.05. (**c**) Apoptosis analysis of TAK-733 (0, 2 μM) in H929 and U266 cells for 48 h representing early and late apoptosis, as well as death population (i), and the effect of treatment for 48 h on apoptosis signaling cleaved caspase 3 and cleaved poly(ADP-ribose) polymerase in H929 cells by Western blotting (ii), (*) *P*< 0.05.

**Figure 2:**
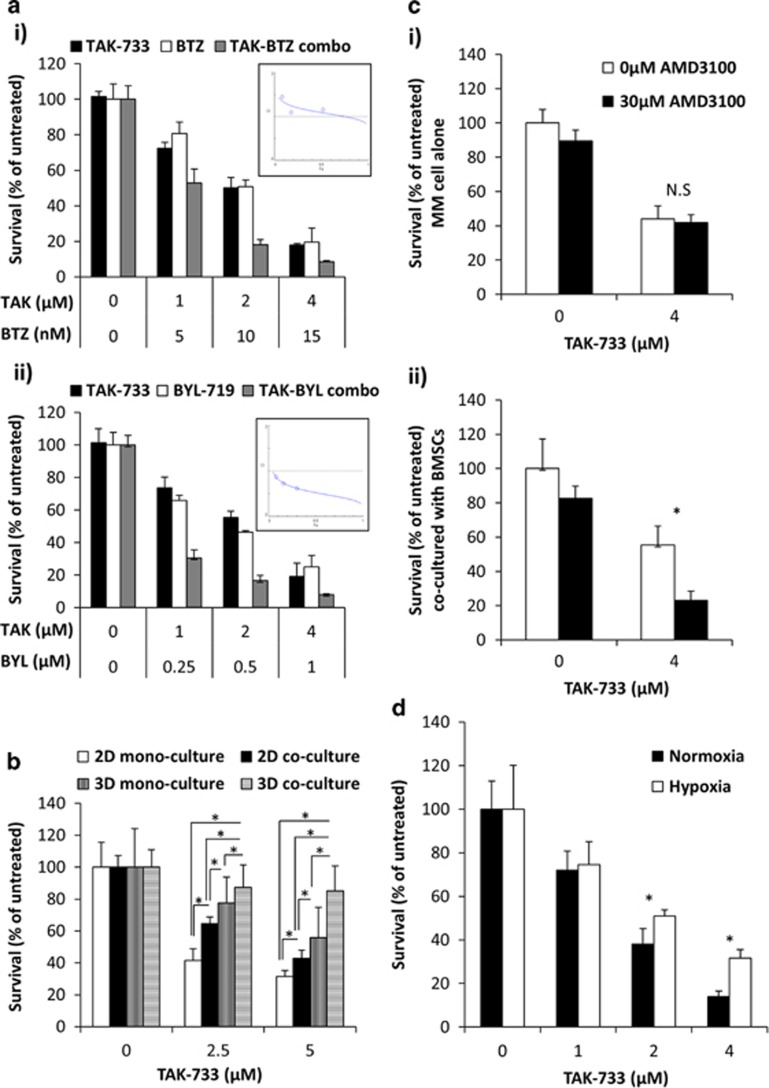
Effect of TAK-733 on drug resistance in the presence of BM microenvironment components. (**a**) Cell viability assay of TAK-733 (0–4 μM) with or without combination of bortezomib (0–15 nM) (i) or BYL-719 (0–1 μM) (ii) for 48 h, in H929 cells; as well CI values to demonstrate synergistic effect of the combination at high concentrations for bortezomib, and at all concentrations for BYL-719 (inserts) measured by the CompuSyn software. (**b**) The effect of TAK-733 (0, 5 μM, 48 h) on survival of MM cell mono-cultures in 2D mono-cultures and co-cultures with BMSCs, compared with 3DTEBM mono-cultures and co-cultures, (*) *P*<0.05. (**c**) The effect of AMD3100 (30 μM) in combination with TAK-733 (4 μM) on survival of MM cells, in case of the absence (i) or presence of BMSCs (ii) after 48 h, (*) *P*<0.05. (**d**) The effect of hypoxia on drug resistance in MM cells shown as the proliferation of MM cells after the treatment with TAK-733 (0–4 μM) for 48 h, analyzed using MTT assay, (*) *P*< 0.05.
